# “Would You Get Vaccinated against COVID-19?” The Picture Emerging from a Study on the Prevalence of SARS-CoV-2 Infection in the General Population of the Veneto Region

**DOI:** 10.3390/vaccines10030365

**Published:** 2022-02-25

**Authors:** Silvia Cocchio, Giulia Tremolada, Patrizia Furlan, Michele Nicoletti, Federico Zabeo, Marco Fonzo, Michele Tonon, Francesca Russo, Vincenzo Baldo

**Affiliations:** 1 Department of Cardiac Thoracic and Vascular Sciences and Public Health, University of Padua, 35131 Padua, Italy; silvia.cocchio@unipd.it (S.C.); giulia.tremolada@studenti.unipd.it (G.T.); patrizia.furlan@unipd.it (P.F.); michele.nicoletti@studenti.unipd.it (M.N.); federico.zabeo@unipd.it (F.Z.); marco.fonzo@unipd.it (M.F.); 2 Regional Directorate of Prevention, Food Safety, Veterinary, Public Health—Veneto Region, 30123 Venezia, Italy; michele.tonon@regione.veneto.it (M.T.); francesca.russo@regione.veneto.it (F.R.)

**Keywords:** COVID-19 vaccine, questionnaire, general population, point of view, vaccine reluctance, vaccine acceptance

## Abstract

COVID-19 disease, caused by the SARS-CoV-2 virus, continues to cause high hospitalization and death rates. Vaccination campaigns have been key to controlling the pandemic, but vaccine hesitancy is on the rise. This study investigated the general population’s attitude to vaccination in Veneto (northeast Italy) in January 2021 as part of a study on the prevalence of SARS-CoV-2 infection. An ad hoc questionnaire collected 4467 respondents’ sociodemographic data and propensity to be vaccinated, and findings were analyzed using logistic multivariable regression. The 48.9% of respondents were male, and the mean age was 46.8 ± 16.0 years. Asked whether they would get vaccinated against COVID-19, 84.3% said yes, 5.0% were uncertain, and 10.7% said no. Vaccine acceptance was higher in males than in females (85.8% vs. 82.8%), in people 70+ years old (92.3%), and among people with more than 14 years of schooling (89.6%). Multivariable analysis with adjOR (95% CI) showed a significantly greater vaccine reluctance in females (0.68 (0.57–0.81)), people 30–49 or 50–69 years old (0.69 (0.54–0.87)), and (0.76 (0.58–0.99)); and those with <9 or 9–13 years of schooling (0.62 (0.46–0.82)), and (0.72 (0.57–0.91)). As people refusing vaccination undeniably hinder efforts to control the pandemic, specific strategies are needed to overcome their doubts.

## 1. Introduction

The COVID-19 pandemic caused by the SARS-CoV-2 virus has prompted a huge number of hospitalization and many deaths. As of 21 December 2021, the World Health Organization (WHO) estimated that there had been 274,628,461 confirmed cases of COVID-19 around the world, and the number of deaths had reached 5,358,978 [1]. The number of countries where COVID-19 is currently present exceeds 215 [2]. From these figures, it is clear that this pandemic will go down in history. The substantial economic and social costs of COVID-19 prompted researchers to seek a safe and effective vaccine as quickly as possible in an effort to minimize the consequences on public health. Once this had been achieved, the general population’s propensity to get vaccinated became a key factor for the success of vaccination campaigns to bring the COVID-19 pandemic safely and effectively under control [3,4]. Vaccine hesitancy can be defined as a reluctance or refusal to be vaccinated once the vaccine has become available [3,5]. It is a growing threat to global health security. In 2019, the WHO included it among the top 10 threats to global public health [5,6,7,8]. There is consensus in the scientific literature that vaccine hesitancy is the result of a complex decision-making process that is influenced by numerous factors, such as complacency, convenience, and confidence, by the context, time, place, and the type of vaccine [8,9,10].

In Italy, the vaccination campaign against SARS-CoV-2 began in January 2021, initially focusing on health workers and then the elderly and very frail. By mid-December 2021, about 73.6% of adults (aged 18 and over) in Italy had completed the vaccination cycle against COVID-19, so more than a quarter (26.4%) of the Italian adult population was still without full coverage [11]. This situation is in line with several studies showing that a significant percentage of the world’s population is reluctant to be vaccinated against COVID-19, and this hesitancy is often seen in specific subpopulations [6,7,8,9,10,11,12]. This makes it essential to establish what type of people are unwilling to be vaccinated against COVID-19 in order to devise tailored strategies to convince them to do so. It is on this portion of the population that a greater commitment is needed to achieve a broader vaccination coverage.

Vaccine hesitancy is currently a rapidly growing phenomenon, so urgent action is needed to increase people’s confidence in the vaccine in order to get more and more people vaccinated and reduce the global impact of the pandemic. Governments and institutions should make a concerted effort to improve communication strategies, nurture confidence in the health system, and achieve greater adherence to the vaccination campaign.

Vaccination campaigns are usually based on multifaceted strategies to increase vaccination coverage, including education/promotion, incentives, organization, and policies. In recent months many governments, including the Italian, have been considering introducing mandatory vaccination policies to increase vaccination coverage among individuals strongly opposed to vaccination against COVID-19. Previous studies have shown that mandatory vaccination policies are effective in achieving the greatest overall vaccination coverage [13]. In Veneto, as elsewhere in Italy, there has been a marked decline in vaccination compliance in recent years, particularly for childhood vaccinations [14]. The Veneto Regional Authority suspended mandatory vaccination in 2007 to contain vaccine hesitancy and counter the spread of antivaccination movements [15]. Italy’s new National Vaccinal Prevention Plan (PNPV 2017–2019), introduced in 2017, provided an extensive offer and active promotion of effective vaccines free of charge, combined with action to support and combat vaccine hesitancy [16]. In July 2017, legislation (Law 119) extended the mandatory vaccination from 4 to 10 vaccines, resulting in a 1% increase in hexavalent childhood vaccination coverage and a 4% increase in MMR vaccination coverage [17].

The aim of this study was to run a survey to investigate the general public’s point of view regarding the vaccination against COVID-19 in view of the campaign’s extension to the population as a whole. Gaining a better idea of people’s different attitudes in relation to their demographic and individual variables could inform vaccination strategies and campaigns to more specifically target the vaccine-hesitant population and thus reduce the proportion of the population refusing vaccination.

## 2. Materials and Methods

### 2.1. Study Population

Data were collected as part of a study on the prevalence of SARS-CoV-2 infection in the general population of the Veneto region (northeast Italy), which had an average population of 4.8 million (8% of the Italian population), with a mean age of 45.4 years, and 51.0% were female. The population’s employment rate was 65.9% [18], and about 21% had more than 14 years of formal education [19]. 

From 8 to 27 January 2021, individuals were recruited on a voluntary basis and had a third-generation rapid antigen test. Participants were drawn from among workers and customers at supermarkets and shopping centers (SSC), Italian Red Cross (IRC) voluntary workers, employees of local authorities (LA), and the Italian Economy and Finance Ministry (MMEF). To be included in the study, they had to be at least 18 years old.

### 2.2. Questionnaire

All participants completed an ad hoc questionnaire (supplementary materials), providing sociodemographic data, their occupation, and their education level. They also gave details about their routine behavior and lifestyle to enable an estimation of the number of their daily contacts (“How many people outside your family do you meet in a day, on average, as part of your job?”), and how many times a day they usually went out (“How often do you leave the house each day?”). Then they answered questions about whether they had been vaccinated against seasonal flu the previous year (“Did you get vaccinated against the flu this year?”) and their attitude to vaccination against COVID-19 (“Would you get vaccinated against COVID-19?”). Referring specifically to COVID-19 vaccination, respondents answering “yes” were named “favorable”, those answering “no” were defined “opposed”, and respondents answering “I don’t Know” were named as “uncertain”.

### 2.3. Data Analysis

The Veneto Regional Authority has developed a centralized regional platform, where the results of all molecular and antigen tests performed to identify SARS-CoV-2 are recorded. For each participant in our study sample, a unique anonymous identification code was generated and used to identify all swabs performed by the same individual prior to their enrollment [20]. The study population was then divided into a “previously screened” group (participants with at least one nasal or nasopharyngeal swab obtained prior to their screening test for the purposes of the study) and a “not previously screened” group.

### 2.4. Statistical Analysis

The data were analyzed using the chi-square test and Student’s *t*-test (for unpaired data), as appropriate. Age was summarized in terms of medians and interquartile ranges (IQR).

Answers to the question “Would you get vaccinated against COVID-19?” were dichotomized, grouping respondents answering “I don’t know” with those answering “No” and defined as “reluctant”, and a multivariable logistic regression was performed to assess participants’ reluctance to undergo COVID-19 vaccination from which adjusted odds ratios (adjORs) and corresponding 95% confidence intervals (95% CI) were calculated. The covariates included in the model were demographic and individual variables, such as sex, age, occupation, education level, routine behavior, and lifestyle. Statistical analyses were performed using SPSS Statistics, version 27.0. A *p* value of less than 0.05 was considered statistically significant.

The sample size was obtained using a prior prevalence for SARS-CoV-2 of 0.4%, a marginal error of 0.2%, and a type 1 error of 5%, two-sided: a minimum sample of 3800 people was needed. The sample size was calculated using EpiInfo Software.

## 3. Results

Between 8 and 27 January 2021, a total of 4467 members of the general population (48.9% of them male) underwent a third-generation rapid antigen test for SARS-CoV-2 and answered our questionnaire. In response to the question “Would you get vaccinated against COVID-19?”, 3765 (84.3%) were favorable, 223 (5.0%) were uncertain, and 479 (10.7%) were opposed (Table 1).

**Figure 1:**
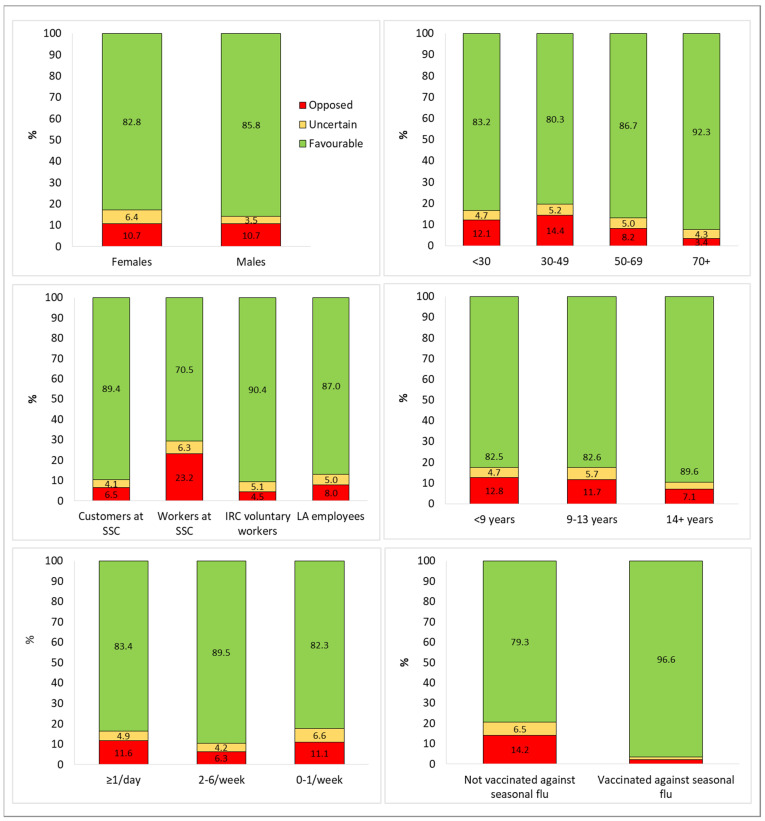
Propensity to be vaccinated against COVID-19, stratified by the main characteristics of the sample.

The mean age of the whole sample was 46.8 ± 16.0 years (median: 48, IQR 25%: 34 and IQR 75%: 59). The opposed group was significantly younger (42.4 ± 13.8 years) than the uncertain group (47.4 ± 16.2 years) or favorable group (46.9 ± 16.4) (*p* < 0.001). The 30- to 49-year-olds were the age group least inclined to get vaccinated, with higher proportions of opposed (14.4%) or uncertain (5.2%). The age groups most favorable to vaccination were over 70 years old (92.3%) or 50–69 (86.7%). As concerns sex, the percentage of favorable respondents was the same among males and females (10.7%), while the percentage of the uncertain was lower for males (3.5%) than for females (6.4%) (Figure 1).

In our sample, 2537 (56.8%) participants were recruited at supermarkets and shopping centers (1481 customers and 1056 workers). These workers accounted for 51.1% of the opposed group and 19.8% of the favorable group (*p* < 0.001). The customers accounted for 20.3% of the opposed group and 35.2% of the favorable group (*p* < 0.001) (Table 1). When propensity to be vaccinated was stratified by participant type, the highest percentages of opposed or uncertain were among the workers at supermarkets and shopping centers (with 23.2% and 6.3%, respectively), whereas the IRC voluntary workers showed the highest propensity to be vaccinated (90.4%) (Figure 1).

Excluding 99 participants (2.2% of the whole sample) who did not indicate their years of schooling, it emerged that people with <14 years of education made up 72.3% of the favorable group, whereas they accounted for significantly higher percentages of the uncertain and opposed groups (82.5% and 82.8%, respectively), (*p* < 0.000) (Table 1). Figure 1 shows that the highest percentage of the opposed to COVID-19 vaccination was recorded among those with <9 years of formal education (12.8%); on the other hand, people with more than 14 years of schooling presented the highest propensity to be vaccinated (89.6%).

When we examined the number of participants’ daily contacts, the people with more than five contacts a day accounted for 70.1% and 62.3% of the opposed and uncertain groups, respectively. The people who reported going out more than once a day made up 78.3% of the opposed group and 70.9% of the uncertain group (Table 1). Figure 1 displays how the highest frequency of opponents was among people going out more than one time/day (11.6%) and among those going out 0–1 time/week (11.1%).

In all, there were 1286 participants who said they had been vaccinated against seasonal flu in the winter of 2020–2021 and accounted for 33% of the favorable group, significantly more than the percentages in the uncertain (7.2%) or opposed (5.8%) groups (*p* < 0.001) (Table 1). Among the vaccinated against seasonal flu, 96.6% were favorable, 1.2% uncertain, and 2.2% opposed the COVID-19 vaccination (Figure 1).

Of the sample as a whole, 37.8% had previously been tested for SARS-CoV-2, and 1.7% reported having previously tested positive for the virus (Table 1).

The multivariable analysis (adjOR (95% CI)) showed that, among the individuals more reluctant to undergo COVID-19 vaccination, there were more females (1.47 (1.24–1.75)) and people aged 30–49 (1.45 (1.14–1.85)) or 50–69 (1.32 (1.01–1.71)), compared with people <30 years old. Taking local authority employees for reference, workers at supermarkets and shopping centers were more reluctant (2.13 (1.67–2.71)), while customers were more in favor of vaccination (0.74 (0.56–0.96)). Regarding the influence of education, the multivariable analysis showed that people with <9 years (1.63 (1.22–2.16)) or 9–13 years (1.39 (1.09–1.77)) of schooling were more vaccine reluctant than those with 14+ of formal education. People who went out more than once a day (1.33 (1.01–1.76)) and those who went out 0–1 time a week (1.78 (1.25–2.53)) and people who had not been vaccinated against seasonal flu (6.14 (4.43–8.53)) were more vaccine reluctant. No associations emerged between people’s propensity to be vaccinated and the number of their daily contacts or any previous screening or testing positive for SARS-CoV-2 (Table 2).

## 4. Discussion

This study examined the willingness to be vaccinated against COVID-19 in a sample of the general population in northeast Italy. The aim was to identify predictors of COVID-19 vaccine reluctance with a view to informing strategies to improve the overall vaccination coverage and thereby reduce the impact of the pandemic on public health.

It is important to emphasize that vaccination propensity was high in this study population, with 84.3% of the sample as a whole in favor of vaccination. This finding is of interest considering that it was obtained from the population at a time when the vaccines had only just become available, and only for selected categories of the population, such as healthcare workers and frail patients [21]. Considering that the factors influencing a population’s vaccination compliance include people’s confidence in institutions and perception of the risk associated with infection (complacency) [8,9,10], our study findings could be interpreted as indicative of the Veneto population showing a good level of trust in the region’s institutions and the recommendations of doctors and the scientific community.

That said, 15.7% of the sample was reluctant to be vaccinated, and 10.7% of participants claimed to be against vaccination. Many governments, including the Italian, are considering introducing mandatory vaccination as an extreme strategy to improve adherence, to increase vaccination coverage even among individuals strongly opposed to vaccination against COVID-19. Previous studies have shown that making vaccination mandatory may induce the hesitant population to be vaccinated to avoid incurring sanctions and may also be interpreted by citizens as a sign that a vaccine is safe [13].

Our results showed that women were significantly less inclined to get vaccinated against COVID-19, in line with previous reports of a positive association between male sex and attitude to COVID-19 vaccination [22,23]. Several studies found the risk of the disease causing severe complications and mortality higher for men than for women [24,25], and some have suggested that this may make men more willing to be vaccinated [22].

Studies that considered the link between age and intention to get vaccinated against COVID-19 generated different findings [12]. Age is thought to be one of the demographic factors most likely to influence people’s propensity to get vaccinated [26]. Our analysis identified two age groups (30–49 and 50–69 years old) as more vaccine reluctant than younger people. The stronger propensity of younger people (<30 years old) in our sample to get vaccinated is in contrast with many other published reports of vaccination acceptance increasing with age [26]. Vaccine hesitancy may stem from young and middle-aged adults considering themselves at lower risk, whereas elderly people are presumably keener to get vaccinated because they feel more exposed to the risk of severe disease and death due to COVID-19 [27]. Another possible explanation why people aged 30 to 70 in our sample were less inclined to get vaccinated could relate to their spending more time on social media than the elderly. Several studies have emphasized a strong connection between social media exposure and skepticism regarding the COVID-19 vaccine and shown how misinformation can be responsible for lower vaccination rates and consequently hinder vaccination coverage [28,29].

We also examined the acceptance of the COVID-19 vaccine by the type of participant enrolled in our sample. Taking local authority employees for reference, it emerged that supermarket and shopping center employees were less inclined to get vaccinated, and customers of large retailers were more likely to do so. Our results concerning supermarket and shopping center employees are consistent with several studies reporting a lower propensity to be vaccinated among people not working in the healthcare sector [30,31]. This difference is probably, at least partly, attributable to a conviction that the threat posed by the pandemic is overrated and that vaccination is unnecessary [32]. However, employees of large retailers are at high risk of infection and COVID-19. A US study found that workers who were in direct contact with customers were five times more likely to become positive for SARS-CoV-2 than workers who were not. It is therefore very important to take action to improve the vaccination coverage in this particular subpopulation [33].

Our data also confirmed the association between education level and vaccine acceptance reported in several scientific articles; people with fewer years of formal education were less likely to get vaccinated [34,35]. Greater adherence to vaccination campaigns among people with higher education may be explained by a stronger awareness of the risks posed by COVID-19 and of the importance of following medical advice.

Our study also showed that vaccine hesitancy featured among people who went out more than once a day and also among those who seldom left their homes (less than once a week). In both cases, this could be due to misconceptions regarding the risk associated with COVID-19. People going out more than once a day were probably not very concerned about the virus and therefore judged vaccination unnecessary. Those going out less than once a week may have felt less exposed to the risk than the rest of the population and therefore believed that there was little advantage in getting vaccinated. In both cases, this would suggest that these people underestimate COVID-19. Several published studies investigated the association between vaccine hesitancy and the perception of the risk posed by the disease [26]. Perceiving the risk as low is one of the most common reasons for refusing vaccination, alongside doubts regarding the vaccine’s efficacy and concern about possible adverse effects [36,37]. Adequate perception of the risk posed by the disease is an essential factor supporting people’s willingness to get vaccinated [36,38].

Our findings also confirmed a positive association between previous seasonal flu vaccinations and the acceptance of COVID-19 vaccination. This is presumably because people who get vaccinated against the flu every year have confidence in vaccination campaigns, are more aware of the importance of prevention in public health, and take more care of their own health than people who do not get vaccinated.

Our study has several limitations. The composition of the study population may not represent the true picture of the Veneto population; the sample was drawn from specific social categories (supermarket customers, supermarket workers, Italian Red Cross volunteers, employees of local authorities, and the Italian Economy and Finance Ministry). Our findings also come from a COVID-19 cross-sectional study, for which individuals were recruited on a voluntary basis, and this may have (i) biased the estimation of vaccination adherence emerging from the questionnaire by comparison with that of the real population and (ii) only provided a snapshot of the population’s response during a limited period of time. People’s intention to be vaccinated against COVID-19 may change in the future, and another limitation related to this study’s design lies in the lack of in-depth information on issues such as the reasons for people’s reluctance to be vaccinated.

Despite the above limitations, the considerable sample size involved means that our study provides some useful information regarding the demographic and individual variables associated with the vaccine-hesitant population.

COVID-19 vaccine reluctance is an increasingly important public health issue and an undeniable obstacle to the achievement of the immunization rates needed to overcome the pandemic. In our sample, the identikit of people unwilling to get vaccinated against COVID-19 had the following characteristics: female, 30–69 years old, and with <14 years of formal education. 

## Figures and Tables

**Table 1:** Characteristics of the study population, stratified by a propensity to be vaccinated against COVID-19.

Characteristics	Favorable n. 3765		Uncertain n. 223		Opposed n. 479		Total n. 4467	
	n.	(%)	n.	(%)	n.	(%)	n.	(%)
Sex								
Males	1875	(49.8)	76	(34.1)	234	(48.9)	2185	(48.9)
Females	1890	(50.2)	147	(65.9)	245	(51.1)	2282	(51.1)
Age group								
<30	661	(17.6)	37	(16.6)	96	(20.0)	794	(17.8)
30–49	1255	(33.3)	82	(36.8)	225	(47.0)	1562	(35.0)
50–69	1548	(41.1)	90	(40.4)	147	(30.7)	1785	(40.0)
70+	301	(8.0)	14	(6.3)	11	(2.3)	326	(7.3)
Type of participant								
Customers at supermarkets and shopping centers	1324	(35.2)	60	(26.9)	97	(20.3)	1481	(33.2)
Workers at supermarkets and shopping centers	745	(19.8)	66	(29.6)	245	(51.1)	1056	(23.6)
Italian Red Cross voluntary workers	443	(11.8)	25	(11.2)	22	(4.6)	490	(11.0)
Local authority employees	1253	(33.3)	72	(32.3)	115	(24.0)	1440	(32.2)
Education level ^								
<9 years	879	(23.8)	50	(23.7)	136	(28.9)	1065	(24.4)
9–13 years	1787	(48.5)	124	(58.8)	253	(53.8)	2164	(49.5)
14+ years	1021	(27.7)	37	(17.5)	81	(17.2)	1139	(26.1)
Daily contacts (other than family)								
No one	421	(11.2)	24	(10.8)	27	(5.6)	472	(10.6)
1–5	1223	(32.5)	60	(26.9)	116	(24.2)	1399	(31.3)
5+	2121	(56.3)	139	(62.3)	336	(70.1)	2596	(58.1)
Number of times they went out								
≥1/day	2686	(71.3)	158	(70.9)	375	(78.3)	3219	(72.1)
2–6/week	642	(17.1)	30	(13.5)	45	(9.4)	717	(16.1)
0–1/week	437	(11.6)	35	(15.7)	59	(12.3)	531	(11.9)
Vaccinated against current seasonal flu	1242	(33.0)	16	(7.2)	28	(5.8)	1286	(28.8)
Previously tested for SARS-CoV-2	1443	(38.3)	83	(37.2)	161	(33.6)	1687	(37.8)
Previously found positive for SARS-CoV-2	63	(1.7)	2	(0.9)	12	(2.5)	77	(1.7)

^ Percentages calculated using the adjusted denominator obtained after excluding participants who did not indicate their education level.

**Table 2:** Multivariable analysis (dependent variable: “Reluctant” to be vaccinated against COVID-19).

	Total	Reluctant		adjOR (IC 95%)
Characteristics	n. 4467	n. 702		
		*n*	(%)	
Sex				
Males	2185	310	(14.2)	Ref
Females	2282	392	(17.2)	1.47 (1.24–1.75)
Age group				
<30	794	133	(16.8)	Ref
30–49	1562	307	(19.7)	1.45 (1.14–1.85)
50–69	1785	237	(13.3)	1.32 (1.01–1.71)
70+	326	25	(7.7)	1.43 (0.86–2.40)
Type of participant				
Customers at supermarkets and shopping centers	1481	157	(10.6)	0.74 (0.56–0.96)
Workers at supermarkets and shopping centers	1056	311	(29.5)	2.13 (1.67–2.71)
Italian Red Cross voluntary workers	490	47	(9.6)	0.74 (0.52–1.06)
Employees of local authorities	1440	187	(13.0)	Ref
Education level				
<9 years	1065	186	(17.5)	1.63 (1.22–2.16)
9–13 years	2164	377	(17.4)	1.39 (1.09–1.77)
14+ years	1139	118	(10.4)	Ref
Daily contacts (other than family)				
No one	472	51	(10.8)	Ref
1–5	1399	176	(12.6)	1.26 (0.88–1.80)
5+	2596	475	(18.3)	1.10 (0.77–1.59)
Number of times they went out				
≥1/day	3219	533	(16.6)	1.33 (1.01–1.76)
2–6/week	717	75	(10.5)	Ref
0–1/week	531	94	(17.7)	1.78 (1.25–2.53)
Vaccinated against current seasonal influenza (no vs. yes)	3181	658	(20.7)	6.14 (4.43–8.53)
Previously screened (no vs. yes)	2780	458	(16.5)	1.07 (0.89–1.28)
Previously tested positive for COVID-19 (no vs. yes)	4390	688	(15.7)	1.23 (066–2.29)

## Data Availability

The data supporting the findings of this study are available from the corresponding author upon reasonable request.

## Supplementary Materials

The following are available online at https://www.mdpi.com/article/10.3390/vaccines10030365/s1, Supplementary file: questionnaire.
