# Supplementary material for: “Would You Get Vaccinated against COVID-19?” The Picture Emerging from a Study on the Prevalence of SARS-CoV-2 Infection in the General Population of the Veneto Region
Source: Vaccines (Basel). 2022 Feb 25;10(3):365. doi: 10.3390/vaccines10030365 (PMC8951628; doi:10.3390/vaccines10030365)
Supplement: Supplementary file 1 [file vaccines-10-00365-s001.zip › vaccines-1594542-supplementary.pdf]

REGIONE DEL VENETO

QUESTIONARIO RACCOLTA INFORMAZIONI

Progetto: "Attività di ricerca al fine di migliorare le attività di prevenzione della Regione Veneto"

(Deliberazione Giunta Regionale del Veneto n. 1643/2020)

Le informazioni verranno utilizzate esclusivamente in forma aggregata e con finalità di ricerca

ID

800 ANNI 1222-2022

UNIVERSITÀ DEGLI STUDI DI PADOVA

**Nome e cognome**	**Sesso**: ☐ maschio ☐ femmina

**Data di nascita** / /	**Provincia di residenza/domicilio abituale**	☐ VENEZIA ☐ PADOVA ☐ ROVIGO	☐ VERONA ☐ BELLUNO ☐ ALTRA	☐ VICENZA ☐ TREVISO

**Codice fiscale**	**Email**	**telefono**

Partecipo in qualità di: ☐ dipendente comune ☐ CRI ☐ GdF ☐ collaboratore grande distribuzione ☐ altro (indicare)

Oppure ☐ figlio ☐ genitore ☐ compagno/a ☐ altro (indicare ____________)

Indicare il nome e cognome della persona di riferimento invitata al progetto

**Indica le Regione dove hai soggiornato nell'ultima settimana** ☐ VENETO<br>☐ Altra regione (indicare)	**Quante sono le persone che vivono con te**<br>☐ 0 ☐ 1 ☐ 2 ☐ 3 ☐ 4 ☐ 5 ☐ >5

**Istruzione**	☐ primaria (elementari)	☐ secondaria secondo grado (superiori)
	☐ secondaria primo grado (medie)	☐ superiore (università)

**Occupazione**	☐ studente	☐ disoccupato	☐ casalinga
	☐ libero professionista	☐ dipendente	☐ docente
	☐ pensionato	☐ altro (specificare)	

**Nell'ambito della tua occupazione, quante persone (al di fuori del tuo nucleo familiare) incontri mediamente in una giornata?**	☐ nessuna
	☐ una
	☐ tra 2 e 5
	☐ tra 6 e 10
	☐ più di 10

Prendi farmaci o ti è stata diagnosticata una patologia cronica

☐ no	☐ si indica quale	☐ malattie del cuore	☐ diabete	☐ pressione alta
		☐ malattie respiratorie	☐ tumore	☐ deficit immunitari
		☐ allergie	☐ malattie renali	

**Hai avuto uno dei seguenti sintomi**	oggi	Settimana scorsa	8-14 giorni fa
Nessuno	☐	☐	☐
Febbre (> 37°)	☐	☐	☐
Mal di gola	☐	☐	☐
Dolore muscolare	☐	☐	☐
Mal di testa	☐	☐	☐
Non sento bene gli odori	☐	☐	☐
Non sento bene i sapori	☐	☐	☐

Ha mai eseguito un tampone? ☐ Mai ☐ Negli ultimi 14 giorni ☐ più di 14 giorni fa

Motivazione ultimo tampone ☐ sintomi ☐ contatto con sospetto e/o positivo ☐ screening

**Sei mai stato in isolamento fiduciario/quarantena** (se non ricordi le date inserire il mese)	☐ no	☐ si	dal<br>al
**Sei mai stato positivo al Covid** (se non ricordi le date inserire il mese)	☐ no	☐ si	dal<br>al

**Al giorno, con quale frequenza esci di casa?**	☐ non esco più
	☐ meno di una volta a settimana
	☐ una volta a settimana
	☐ 2-6 volte a settimana
	☐ una volta al giorno
	☐ più volte al giorno

**Ti vaccineresti contro il Covid?**	☐ no	☐ si	☐ non so
**Ti sei vaccinato contro l'influenza quest'anno?**	☐ no	☐ si	

**Esito tampone (a cura del laboratorio)**	ID Pool	☐ positivo	☐ negativo
